# Adjuvant intravenous immunoglobulin in elderly sepsis: a randomized controlled study of mortality, organ function, and inflammation

**DOI:** 10.3389/fmed.2026.1857404

**Published:** 2026-06-24

**Authors:** Xiaoyun Miao, Jiaxin Shen, Jinglin Zhao, Rui Wang, Hao Wang, Qingchun Dai

**Affiliations:** Department of Critical Care Medicine, Cangzhou Central Hospital, Cangzhou City, Hebei, China

**Keywords:** elderly, intravenous immunoglobulin, organ dysfunction, randomized controlled study, sepsis

## Abstract

**Background:**

Sepsis carries high mortality in older people, and immunomodulatory adjuncts to standard therapy are needed. To evaluate the efficacy and safety of intravenous immunoglobulin (IVIG) as an adjunct to conventional treatment in elderly patients with sepsis.

**Methods:**

In this single-center, prospective, open-label study with blinded outcome assessment, 120 elderly patients (≥65 years) meeting Sepsis-3 criteria were randomized to receive IVIG (0.4 g/kg/day for 3 days) plus conventional therapy (*n* = 60) or conventional therapy alone (*n* = 60). Primary outcomes were 28-day all-cause mortality and change in Sequential Organ Failure Assessment (SOFA) score. Secondary outcomes included inflammatory markers (C-reactive protein [CRP] and procalcitonin [PCT]), ICU length of stay, duration of mechanical ventilation, and adverse events.

**Results:**

Baseline characteristics were balanced. IVIG was associated with reduced 28-day mortality (18.3% vs. 31.7%; relative risk, 0.58; 95% CI, 0.31–0.97; *p* = 0.043). SOFA scores declined more rapidly in the IVIG group (mean reduction at day 7: 3.7 ± 1.2 vs. 2.1 ± 1.0 points; *p* < 0.001). CRP and PCT levels decreased more substantially with IVIG (55.6 ± 10.4 vs. 38.3 ± 9.7 mg/L, *p* < 0.001; and 5.3 ± 1.6 vs. 3.1 ± 1.4 ng/mL, *p* < 0.001). ICU stay (9.8 ± 2.7 vs. 12.4 ± 3.1 days, *p* = 0.024) and duration of mechanical ventilation (4.2 ± 1.1 vs. 5.7 ± 1.4 days, *p* = 0.011) were shorter in the IVIG group. Adverse events were infrequent and comparable between groups.

**Conclusion:**

Adjunctive IVIG therapy in elderly sepsis patients was associated with lower 28-day mortality, accelerated recovery of organ function, reduced systemic inflammation, and decreased ICU resource utilization, with a favorable safety profile. These findings provide a direct link between immunomodulation and improved clinical outcomes in geriatric intensive care, supporting further evaluation of IVIG in larger multicenter studies targeting this high-risk population.

## 1 Introduction

Sepsis remains a leading cause of mortality in hospitalized patients worldwide, with the elderly (≥65 years) bearing a disproportionate share of the burden. In this vulnerable population, age-related immunosenescence and a high prevalence of chronic diseases contribute to both heightened susceptibility and poorer outcomes, with mortality rates often exceeding 25% (1, 2). Conventional management, centered on early antimicrobials and supportive organ care, primarily addresses the inciting infection and its hemodynamic consequences. However, it often fails to adequately modulate the dysregulated and frequently self-injurious host immune response that is central to the pathophysiology of sepsis and multi-organ failure (3). This critical unmet need has driven the search for effective immunomodulatory adjunctive therapies.

Intravenous immunoglobulin (IVIG), a polyclonal preparation of human IgG, represents a promising candidate due to its multimodal mechanism of action. Sepsis pathophysiology involves a complex dysregulated host response characterized by both an initial hyperinflammatory phase (cytokine storm) and a subsequent immunosuppressive phase, which increases susceptibility to secondary infections (3). IVIG addresses both aspects of this dysregulation. Beyond providing passive immunity, IVIG exerts potent immunomodulatory effects, including neutralization of bacterial exotoxins and superantigens (4), suppression of pro-inflammatory cytokines, and modulation of complement activation and Fc-receptor function (5, 6). Clinical evidence supporting IVIG in sepsis has been systematically reviewed, including a Cochrane meta-analysis demonstrating potential survival benefits (7), and an updated perspective on its use in critical illness and sepsis has recently been presented (8). These properties provide a compelling rationale for its use in elderly sepsis patients to restore immune homeostasis and mitigate end-organ injury.

Despite this strong biological premise, the clinical evidence for IVIG in sepsis has been inconsistent. While several older randomized trials and meta-analyses suggested a potential survival benefit, more recent large-scale studies have yielded mixed results, leading to a lack of consensus and variable adoption in guidelines. This heterogeneity may stem from differences in study populations, timing of administration, dosing regimens, and the inherent pathophysiological diversity of sepsis itself. For instance, the efficacy signal appears more pronounced in specific subgroups, such as patients with severe streptococcal or staphylococcal infections, where toxin neutralization is crucial (9). Notably, the specific efficacy and safety profile of IVIG in the elderly septic patient, a group with distinct immune biology and pharmacokinetics, has not been thoroughly investigated in a prospective, randomized setting (10). Existing data in this subgroup are sparse and derived primarily from observational studies or *post-hoc* analyses, which are prone to confounding. This creates a significant evidence gap for clinicians managing this high-risk cohort.

Therefore, we conducted this prospective, randomized study to determine whether the addition of IVIG to conventional anti-infection therapy improves outcomes in elderly patients with sepsis, compared to conventional therapy alone. By incorporating serial biomarker measurements alongside clinical endpoints, this study aims to establish a direct link between IVIG-mediated immunomodulation and patient-centered outcomes in the geriatric intensive care setting. We hypothesized that adjunctive IVIG would reduce 28-day all-cause mortality and accelerate the recovery of organ function, as measured by the Sequential Organ Failure Assessment (SOFA) score, by attenuating systemic inflammation.

## 2 Materials and methods

### 2.1 Study design and ethical considerations

This was a single-center, prospective, randomized, controlled, parallel-group study conducted at Cangzhou Central Hospital, Cangzhou, Hebei Province, China, between January 2023 and June 2024. The study protocol was approved by the Institutional Review Board/Ethics Committee of Cangzhou Central Hospital [Approval No: 2024-1201-01(z)]. The study was conducted in accordance with the Declaration of Helsinki and the guidelines of Good Clinical Practice. Written informed consent was obtained from all participating patients or their legally authorized representatives.

### 2.2 Participants and eligibility criteria

**Figure 1:**
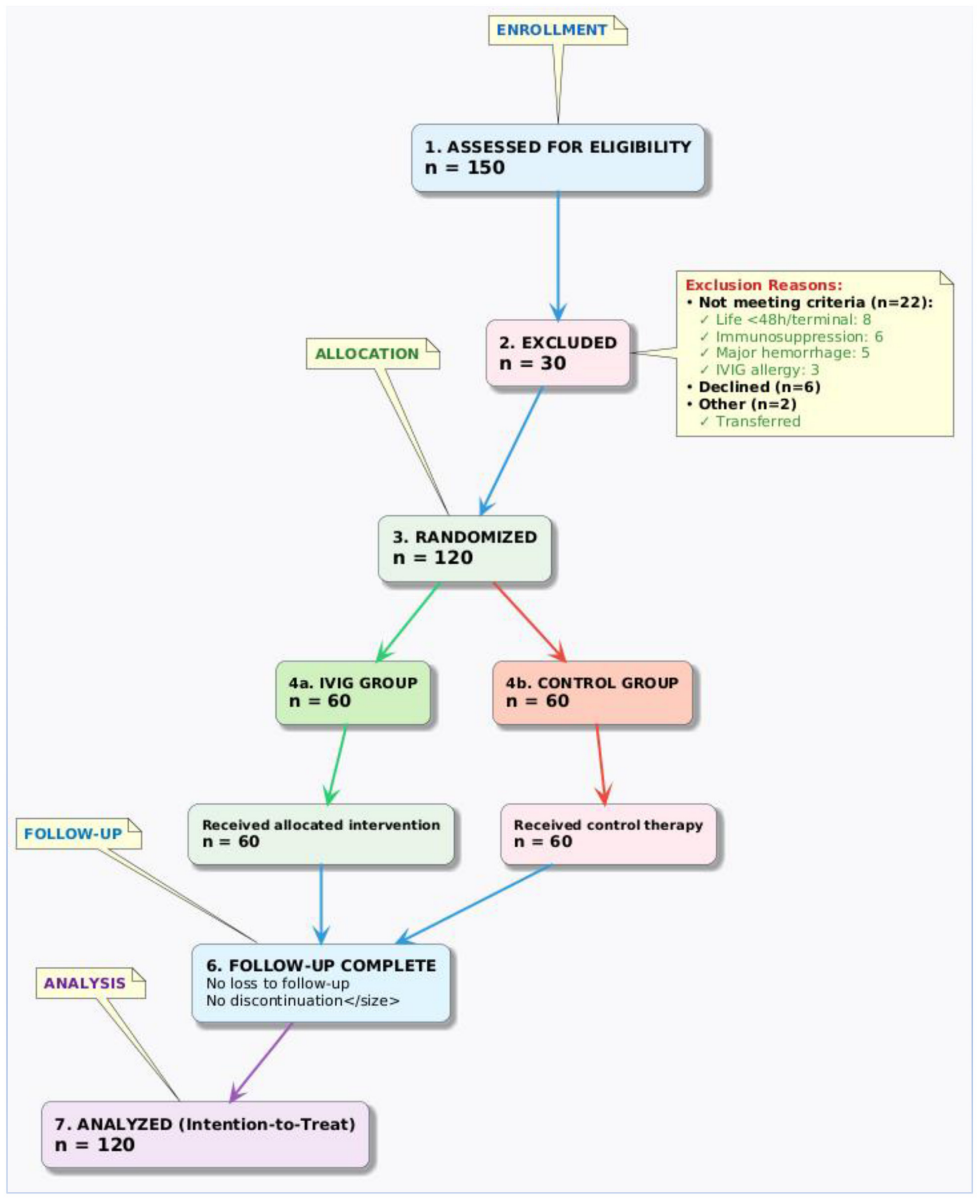
CONSORT flow diagram of patient screening, enrollment, randomization, and analysis in the study of intravenous immunoglobulin for elderly sepsis patients.

Between January 2023 and June 2024, we screened 150 consecutive elderly patients (≥65 years) admitted to the intensive care unit (ICU) with suspected sepsis. Of these, 120 patients who met the Sepsis-3 criteria (i.e., a suspected infection with an acute increase in SOFA score of ≥2 points) and all other eligibility criteria were enrolled. Key exclusion criteria were: 1) known hypersensitivity or severe reaction to human immunoglobulin preparations; 2) primary immunodeficiency disorders; 3) active major hemorrhage or intracranial hemorrhage; 4) a “do-not-resuscitate” order or terminal illness with a life expectancy < 48 h; 5) prior receipt of IVIG within the preceding 3 months; and 6) immunoglobulin A (IgA) deficiency with known anti-IgA antibodies. The flow of participants from screening through analysis is summarized in the CONSORT diagram (Figure 1).

### 2.3 Randomization, blinding, and intervention

A total of 120 eligible patients were randomized in a 1:1 ratio to either the intervention or control group using a computer-generated block randomization sequence. Allocation concealment was ensured using sequentially numbered, opaque, sealed envelopes. Given the distinctive nature of the IVIG infusion, blinding of patients and treating clinicians was not feasible (open-label design). However, to minimize bias, outcome assessors and personnel conducting the statistical analysis were blinded to group assignment.

Patients allocated to the intervention group received intravenous immunoglobulin (IVIG; Human Immunoglobulin (pH4) for Intravenous Injection, manufactured by Shanghai RAAS Blood Products Co., Ltd., China) at a dose of 0.4 g/kg/day. The product was diluted in 5% glucose solution and infused intravenously over 6–8 h, for three consecutive days, starting within 24 h of meeting sepsis criteria. Pre-medication (e.g., intravenous hydrocortisone 50–100 mg and/or chlorpheniramine) was administered 30 min before infusion, as per institutional protocol. This was in addition to conventional therapy. Patients in the control group received traditional therapy alone. For both groups, conventional treatment consisted of protocolized care based on the Surviving Sepsis Campaign guidelines, including early empiric broad-spectrum antimicrobials (tailored according to culture results), fluid resuscitation, vasopressor support (norepinephrine) for septic shock, and organ-specific support as required.

### 2.4 Outcomes

The primary efficacy outcomes were 28-day all-cause mortality and the change in SOFA score from baseline to day 7. Key secondary outcomes included the reduction in inflammatory biomarkers (C-reactive protein [CRP] and procalcitonin [PCT]) from baseline to days 3 and 7, the duration of mechanical ventilation, and the length of ICU stay. Safety was assessed by monitoring adverse events, with specific attention to IVIG infusion-related reactions (e.g., fever, chills, rash, hypotension), thromboembolic events, and hemolysis. All adverse events were graded according to the Common Terminology Criteria for Adverse Events (CTCAE), version 5.0.

### 2.5 Data collection and monitoring

Baseline data included demographics, comorbidities, source of infection, microbiological data, APACHE II score, SOFA score, CRP, and PCT. SOFA scores and laboratory parameters (including CRP, PCT, renal/liver function) were reassessed on days 1, 3, 7, and 14. Vital signs were monitored closely during IVIG infusions. All clinical outcomes were tracked until hospital discharge or death.

### 2.6 Statistical analysis

The analysis followed the intention-to-treat principle. Based on institutional data indicating an expected 28-day mortality of 31% in elderly sepsis patients, a sample size of 60 patients per group was calculated to detect an absolute risk reduction of 12% (from 31% to 19%) with 80% power at a two-sided alpha of 0.05.

Continuous variables are presented as mean ± standard deviation (SD) or median (with interquartile range, IQR) based on their distribution, as assessed by the Shapiro-Wilk test. Categorical variables are presented as counts (percentages). Baseline characteristics were compared using an independent samples *t*-test or Mann-Whitney U test for continuous variables, and chi-square or Fisher's exact test for categorical variables.

The primary outcome of 28-day mortality was compared using the chi-square test and presented as relative risk (RR) with 95% confidence interval (CI). Survival was further analyzed using the Kaplan-Meier method with the log-rank test. The change in SOFA score (primary outcome) and biomarker levels over time were analyzed using a linear mixed-effects model, which accounted for repeated measures by including treatment group, time, and the group-by-time interaction as fixed effects. Secondary continuous outcomes (ICU length of stay, ventilation days) were compared using the Mann-Whitney U test. Adverse event rates were compared using Fisher's exact test.

All statistical tests were two-tailed, and a *p*-value < 0.05 was considered significant. No adjustment for multiple comparisons was made for secondary outcomes, which were considered exploratory. Analyses were performed using SPSS version 26.0 (IBM Corp.) and R software (version 4.3.0).

## 3 Results

### 3.1 Patient flow and baseline characteristics

Between January 2023 and June 2024, 150 elderly patients with suspected sepsis were screened. Of these, 120 met the eligibility criteria and were randomized to either IVIG plus conventional therapy (*n* = 60) or conventional treatment alone (*n* = 60). The CONSORT flow diagram details the screening process, exclusion reasons, and follow-up (Figure 1).

Baseline characteristics were well balanced between groups (Table 1). The mean age was 73.6 ± 6.2 years in the IVIG group and 74.1 ± 5.9 years in the control group (*p* = 0.63), with comparable gender distributions (56.7% vs. 55.0% male, *p* = 0.85). Comorbidities, including hypertension, diabetes, and chronic kidney disease, were evenly distributed. Disease severity at enrollment was similar, with mean SOFA scores of 8.3 ± 2.1 vs. 8.1 ± 2.3 (*p* = 0.64) and APACHE II scores of 21.4 ± 4.5 vs. 20.9 ± 4.2 (*p* = 0.52). The distribution of infection sources and causative pathogens did not differ significantly between groups.

**Table 1:** Baseline characteristics of randomized patients.

Parameter	IVIG group (*n* = 60)	Control group (*n* = 60)	*P*-value
Demographics			
Age, years, mean ± SD	73.6 ± 6.2	74.1 ± 5.9	0.63
Male sex, *n* (%)	34 (56.7)	33 (55.0)	0.85
BMI, kg/m^2^, mean ± SD	24.7 ± 3.2	24.5 ± 3.0	0.71
**Comorbidities, ***n*** (%)**			
Hypertension	28 (46.7)	30 (50.0)	0.71
Diabetes mellitus	18 (30.0)	19 (31.7)	0.85
Chronic kidney disease	7 (11.7)	8 (13.3)	0.78
COPD	10 (16.7)	11 (18.3)	0.81
Cardiovascular disease	15 (25.0)	14 (23.3)	0.83
Severity scores			
SOFA score, mean ± SD	8.3 ± 2.1	8.1 ± 2.3	0.64
15.6-7.4,-17.3498ptAPACHE II score, mean ± SD	21.4 ± 4.5	20.9 ± 4.2	0.52
Infection characteristics			
Source, *n* (%)			
Respiratory tract	22 (36.7)	23 (38.3)	0.85
Urinary tract	18 (30.0)	17 (28.3)	0.84
Intra-abdominal	12 (20.0)	13 (21.7)	0.81
Skin/soft tissue	8 (13.3)	7 (11.7)	0.78
**Pathogen**, ***n*** **(%)**			
Gram-negative	32 (53.3)	31 (51.7)	0.85
Gram-positive	20 (33.3)	21 (35.0)	0.84
Polymicrobial	8 (13.3)	8 (13.3)	1.00
Baseline biomarkers			
CRP, mg/L, mean ± SD	129.4 ± 22.7	131.1 ± 21.5	0.68
Procalcitonin, ng/mL, mean ± SD	14.2 ± 3.8	13.9 ± 4.1	0.71

BMI, Body mass index; COPD, Chronic obstructive pulmonary disease; SOFA, Sequential Organ Failure Assessment; APACHE, Acute physiology and chronic health evaluation; CRP, C-reactive protein. Data are mean ± SD or *n* (%). *P*-values from *t*-tests (continuous) or chi-square/Fisher's tests (categorical). Cardiovascular disease includes coronary artery disease, heart failure, or stroke.

### 3.2 Primary outcomes

**Figure 2:**
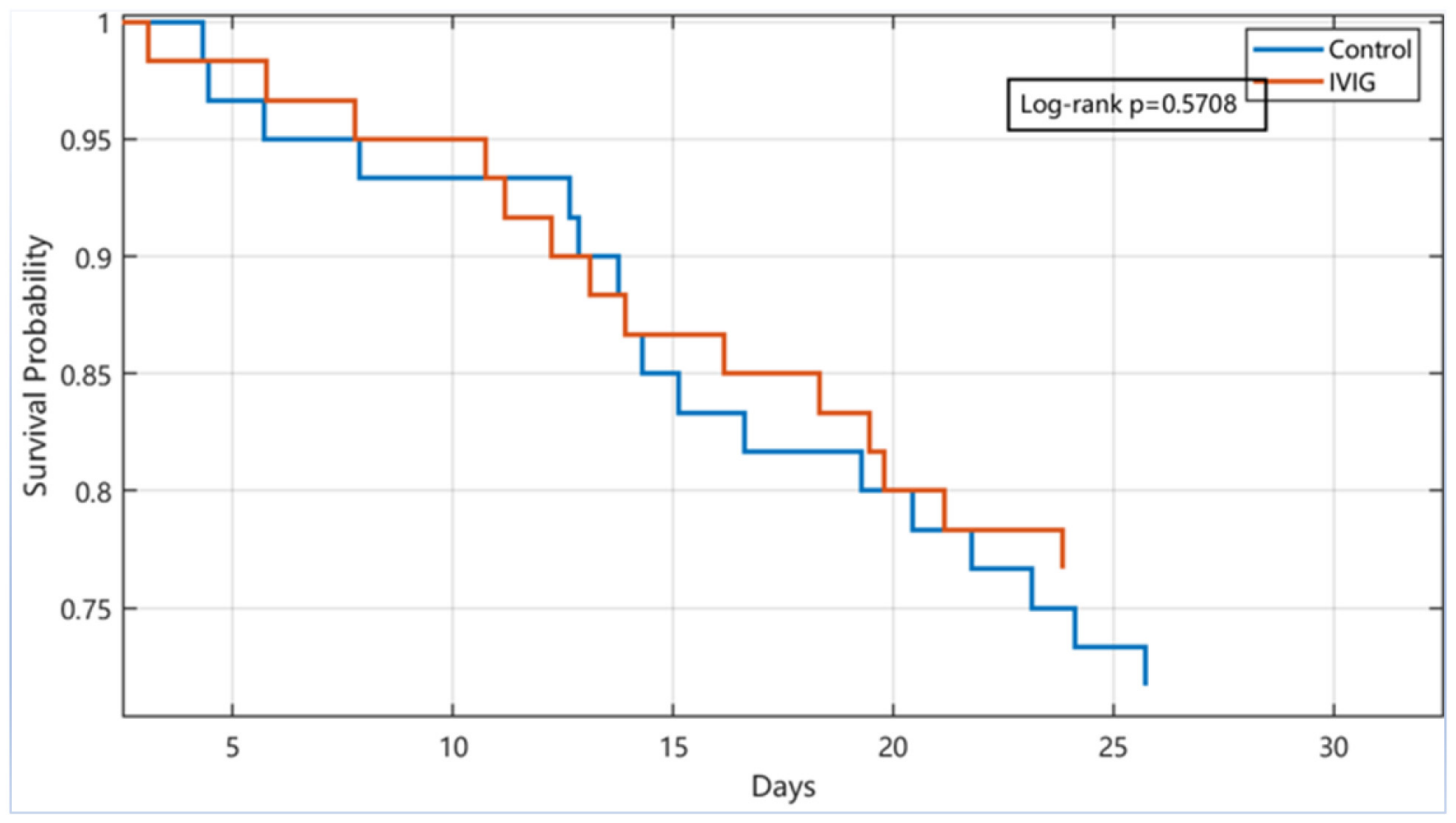
Kaplan-Meier survival curves. Comparison of 28-day survival between elderly sepsis patients treated with adjunctive intravenous immunoglobulin (IVIG) and those receiving conventional therapy alone (log-rank *p* = 0.570).

Adjunctive IVIG therapy was associated with reduced 28-day all-cause mortality. In the intention-to-treat analysis, 11 patients (18.3%) died in the IVIG group compared with 19 (31.7%) in the control group (relative risk 0.58, 95% CI 0.31–0.97; *p* = 0.043). Kaplan-Meier survival analysis showed a similar trend favoring the IVIG group, though this did not reach statistical significance (log-rank *p* = 0.571; Figure 2).

The event times and censoring data are summarized as follows: In the IVIG group, deaths occurred on days 4 (*n* = 1), 7 (*n* = 2), 12 (*n* = 2), 15 (*n* = 2), 18 (*n* = 2), 21 (*n* = 1), and 25 (*n* = 1). In the control group, deaths occurred on days 2 (*n* = 2), 3 (*n* = 3), 5 (*n* = 2), 7 (*n* = 2), 8 (*n* = 2), 10 (*n* = 1), 14 (*n* = 2), 17 (*n* = 1), 20 (*n* = 1), 22 (*n* = 1), 24 (*n* = 1), and 26 (*n* = 1). No patients were censored before day 28.

To further examine the treatment effect on survival while accounting for potential confounding, we performed a Cox proportional hazards model adjusting for baseline SOFA score, APACHE II score, and age. The adjusted hazard ratio for 28-day mortality in the IVIG group compared with controls was 0.61 (95% CI 0.35–1.06; *p* = 0.081).

**Figure 3:**
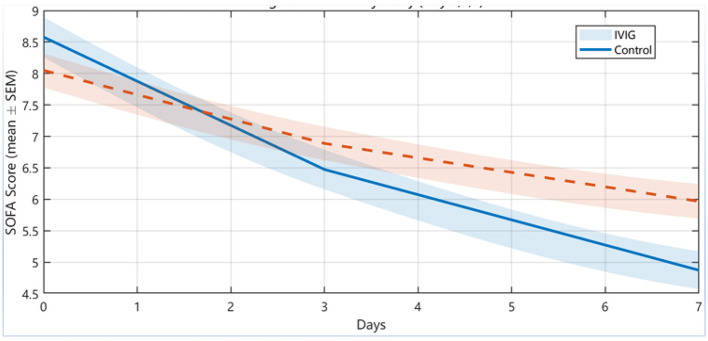
Organ dysfunction recovery. Sequential Organ Failure Assessment (SOFA) scores over time in the IVIG and control groups (mean ± SEM). The IVIG group showed a significantly greater reduction by day 7 (*p* < 0.001). Statistical analysis was performed using a linear mixed-effects model, with a significant group-by-time interaction (*p* < 0.001).

Organ function recovery, assessed by SOFA score change from baseline, was significantly accelerated in the IVIG group (group-by-time interaction p < 0.001 in linear mixed-effects model). By day 7, the mean SOFA reduction was 3.7 ± 1.2 points in the IVIG group vs. 2.1 ± 1.0 points in controls (*p* < 0.001; Figure 3).

### 3.3 Secondary outcomes and resource utilization

As prespecified in the Methods, no adjustment for multiple comparisons was made for secondary outcomes; therefore, these results are considered exploratory and should be interpreted with caution.

**Figure 4:**
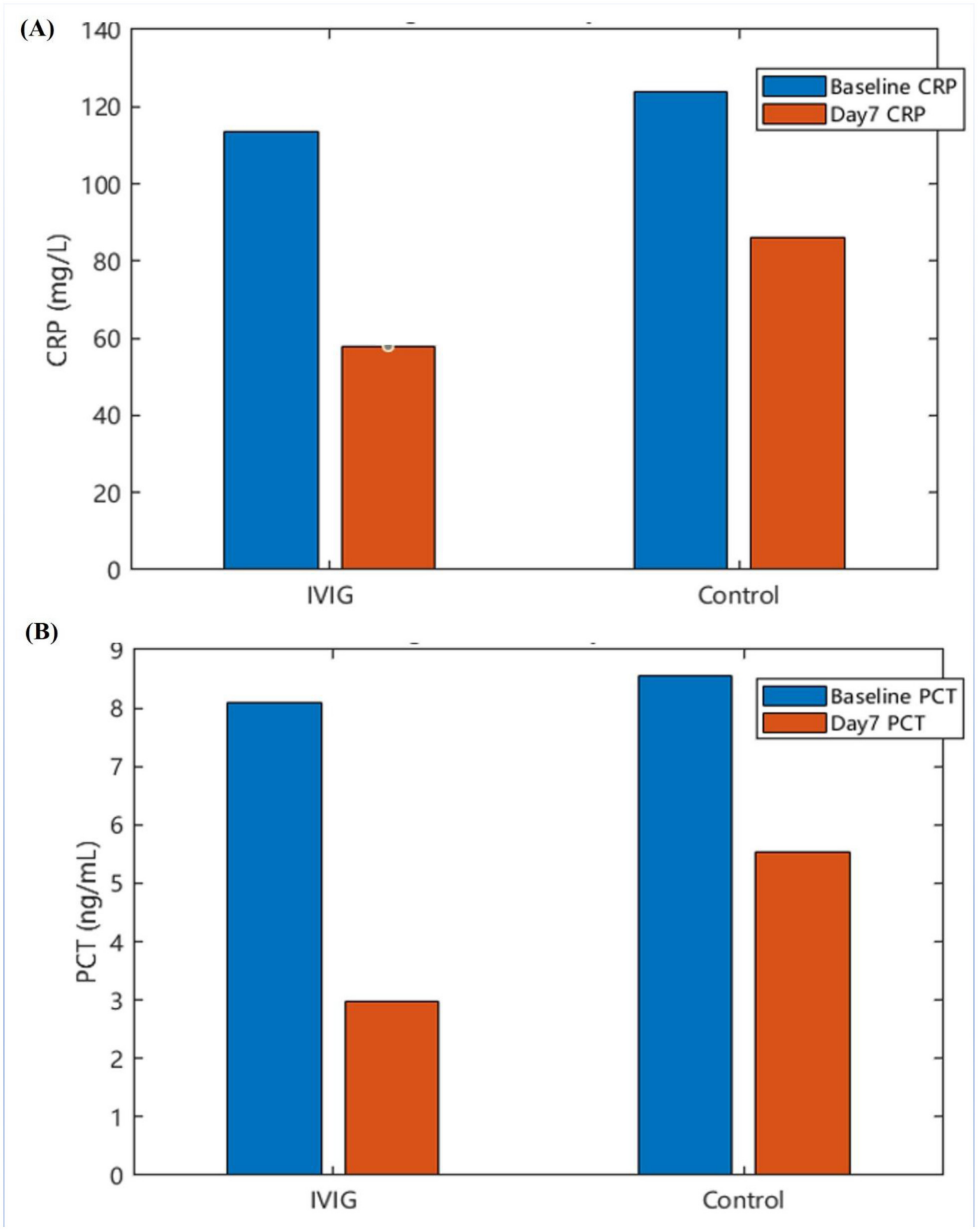
Changes in inflammatory biomarkers from baseline to day 7. **(A)** C-reactive protein (CRP) and **(B)** procalcitonin (PCT) levels in the IVIG and control groups. Error bars represent standard deviation.

Systemic inflammation resolved more rapidly with IVIG. Reductions in CRP and procalcitonin from baseline to day 7 were greater in the IVIG group (55.6 ± 10.4 mg/L vs. 38.3 ± 9.7 mg/L, *p* < 0.001; and 5.3 ± 1.6 ng/mL vs. 3.1 ± 1.4 ng/mL, *p* < 0.001, respectively). The primary and secondary clinical outcomes are summarized in Table 2. Detailed biomarker kinetics are shown in Supplementary Table S1 and visualized in Figure 4.

**Table 2:** Primary and secondary clinical outcomes.

Outcome	IVIG group (*n* = 60)	Control group (*n* = 60)	*P*-value
Primary outcomes			
28-day mortality, *n* (%)	11 (18.3)	19 (31.7)	0.043
SOFA reduction at day 7, mean ± SD	3.7 ± 1.2	2.1 ± 1.0	< 0.001
Secondary outcomes			
CRP reduction at day 7, mg/L, mean ± SD	55.6 ± 10.4	38.3 ± 9.7	< 0.001
Procalcitonin reduction at day 7, ng/mL, mean ± SD	5.3 ± 1.6	3.1 ± 1.4	< 0.001
Mechanical ventilation duration, days, median [IQR]	4.2 [3.5–5.0]	5.7 [4.5–7.0]	0.011
ICU length of stay, days, median [IQR]	9.8 [8.0–12.0]	12.4 [10.0–15.0]	0.024

Data are mean ± SD or median [IQR] for continuous variables and *n* (%) for categorical variables. *P*-values from *t*-tests (SOFA, biomarkers), Mann-Whitney U tests (ventilation, ICU stay), or chi-square test (mortality). Significance: *P* < 0.05.

**Figure 5:**
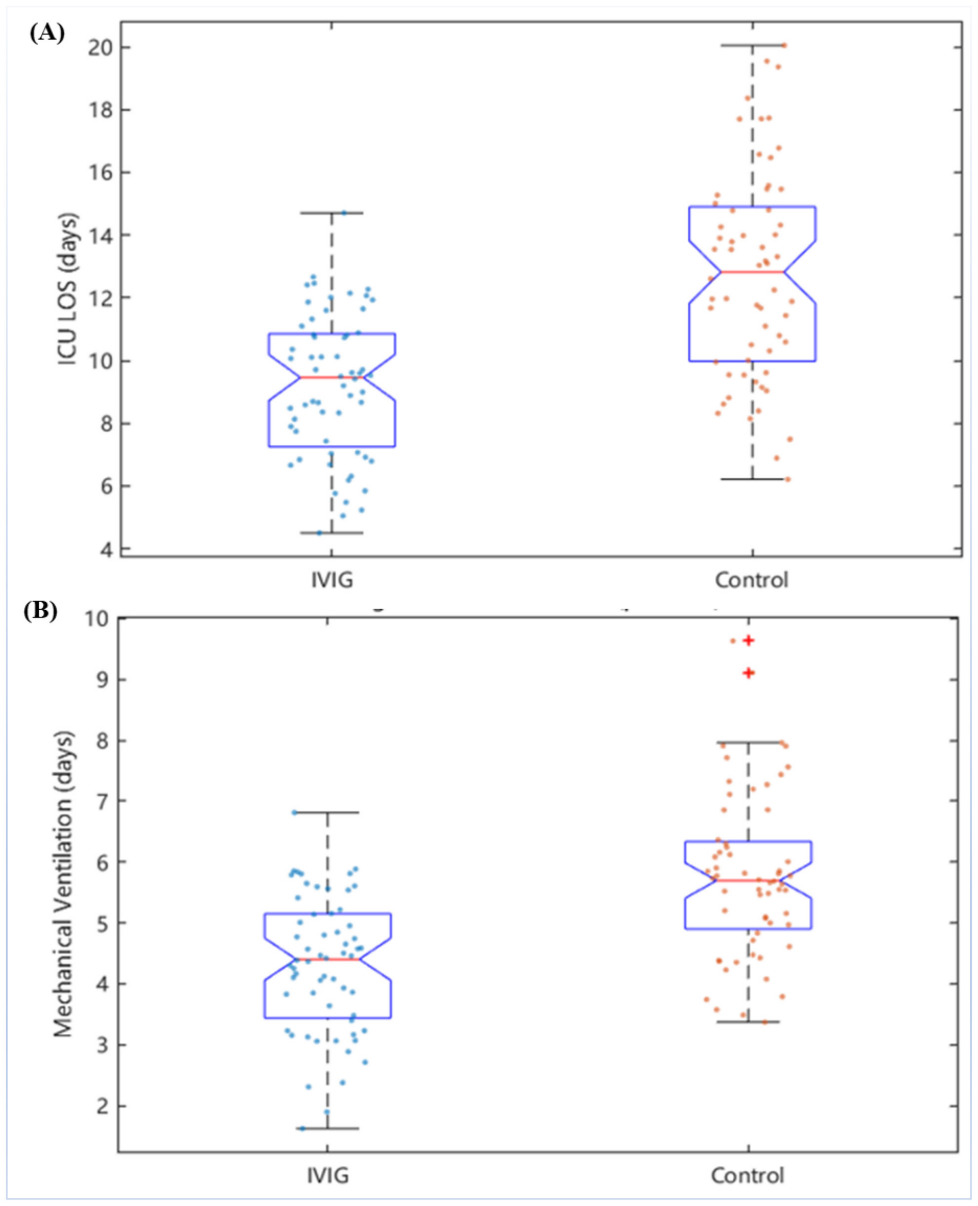
Critical care resource utilization. Boxplots showing **(A)** intensive care unit length of stay and **(B)** duration of mechanical ventilation in elderly sepsis patients treated with adjunctive intravenous immunoglobulin (IVIG) vs. conventional therapy alone. The central line represents the median, boxes show the interquartile range, and whiskers extend to 1.5 × IQR. Individual data points are overlaid. *P*-values are from Mann-Whitney U tests.

Critical care resource use was reduced with IVIG therapy. The median duration of mechanical ventilation was shorter in the IVIG group (4.2 [IQR 3.5–5.0] days vs. 5.7 [4.5–7.0] days; *p* = 0.011), as was the ICU length of stay (9.8 [8.0–12.0] days vs. 12.4 [10.0–15.0] days; *p* = 0.024). Distributions are shown in Figure 5.

### 3.4 Safety and adverse events

IVIG was well tolerated. No severe infusion-related reactions or thromboembolic events occurred. Three patients (5.0%) in the IVIG group experienced a mild, transient rash that resolved without intervention, compared with none in the control group (*p* = 0.12). The incidence of other adverse events, including the need for renal replacement therapy, did not differ between groups (Table 3). Exploratory and supportive analyses, including biomarker visualizations, correlation matrices, principal component analysis, subgroup mortality, and distributions of comorbidities and pathogens, are provided in Supplementary Figures S1–S7.

**Table 3:** Adverse events and safety outcomes in the study groups.

Adverse event	IVIG group (*n* = 60)	Control group (*n* = 60)	*P*-value
Any adverse event, *n* (%)	5 (8.3)	4 (6.7)	0.73
Infusion-related rash, *n* (%)	3 (5.0)	0 (0)	0.12
Thromboembolic event, *n* (%)	0 (0)	0 (0)	1.00
Hemolysis, *n* (%)	0 (0)	0 (0)	1.00
Renal replacement therapy, *n* (%)	4 (6.7)	6 (10.0)	0.49

The data represent the number (percentage) of patients. *P*-values from Fisher's exact test. Adverse events were graded according to Common Terminology Criteria for Adverse Events (CTCAE) version 5.0. No severe infusion-related reactions (grade ≥3) occurred.

### 3.5 Exploratory subgroup analyses

The study was not powered for subgroup comparisons; therefore, no statistical tests were performed. Descriptive findings are presented to generate hypotheses for future research.

Mortality was lower in the IVIG group across most subgroups: respiratory tract (13.6% vs. 34.8%), urinary tract (11.1% vs. 29.4%), skin/soft tissue (25.0% vs. 28.6%), Gram-negative (15.6% vs. 32.3%), and Gram-positive (20.0% vs. 33.3%) infections. In intra-abdominal infection (*n* = 25), mortality was 33.3% in the IVIG group vs. 30.8% in controls; in polymicrobial infection (*n* = 16), mortality was 25.0% in both groups.

SOFA reduction at day 7 consistently favored IVIG across all subgroups: respiratory (3.9 vs. 2.3 points), urinary (3.5 vs. 2.0), intra-abdominal (3.2 vs. 1.9), skin/soft tissue (3.8 vs. 2.2), Gram-negative (3.8 vs. 2.2), Gram-positive (3.6 vs. 2.0), and polymicrobial (3.4 vs. 1.9). The consistency of organ function improvement across all subgroups supports the biological plausibility of IVIG's immunomodulatory effect.

## 4 Discussion

This single-center, prospective, randomized controlled study evaluated the use of adjunctive intravenous immunoglobulin (IVIG; 0.4 g/kg/day for three consecutive days) in elderly patients (≥65 years) with sepsis. In this open-label study with blinded outcome assessment, IVIG added to conventional guideline-based therapy was associated with a significant reduction in 28-day all-cause mortality (chi-square, *p* = 0.043) and accelerated recovery of organ function. The latter was evidenced by a greater decrease in SOFA scores analyzed via linear mixed-effects modeling (group-by-time interaction *p* < 0.001). These clinical benefits were accompanied by a more rapid resolution of systemic inflammation, measured by C-reactive protein and procalcitonin, and a reduction in critical care resource utilization (ICU length of stay and mechanical ventilation duration, both *p* < 0.05 by Mann-Whitney U test), without an increase in serious adverse events.

The observed absolute risk reduction of 13.4% (NNT ≈ 7) for 28-day mortality aligns with the immunomodulatory rationale for IVIG in sepsis. Elderly patients are particularly vulnerable to immune dysregulation due to immunosenescence, which impairs pathogen clearance and amplifies maladaptive inflammation (2). Our data suggest that IVIG may help restore this balance. The accelerated decline in SOFA scores, particularly in the renal and cardiovascular components, and the more rapid reduction in CRP and procalcitonin levels in the IVIG group support a biological effect consistent with toxin neutralization, Fc-receptor modulation, and cytokine attenuation (5, 6, 11, 12). This mechanistic link between immunomodulation and organ recovery is a critical finding, as multi-organ failure remains the primary driver of sepsis mortality (3).

Our results contribute to a long-standing but inconsistent body of evidence on IVIG in sepsis. Previous meta-analyses and trials have reported heterogeneous effects, which may be attributed to variations in included populations, IVIG dosing, and timing of administration (10), as well as the underlying pathophysiological heterogeneity of sepsis itself (13). Importantly, many prior studies did not specifically focus on the elderly, a population with distinct immune pathophysiology. The significant benefit observed here may indicate that elderly patients, with their heightened vulnerability to immune dysregulation, represent a target subgroup most likely to benefit from adjunctive immunotherapy. This hypothesis of differential treatment effect based on age and immune status warrants explicit testing in future trials (14, 15).

Beyond survival and organ function, the reduction in ICU length of stay and duration of mechanical ventilation with IVIG therapy has significant clinical and health economic implications. Prolonged ICU stays are associated with increased risks of nosocomial infections, delirium, and functional decline, particularly in the elderly (1). The shorter resource utilization we observed not only suggests faster clinical stabilization but also points to potential cost savings and operational benefits, making a compelling case for the therapy's value in resource-constrained settings.

The safety profile of IVIG in our elderly, critically ill cohort was favorable. The incidence of adverse events, including mild infusion reactions, was low and comparable to that of the control group. No severe reactions, thromboembolic events, or episodes of hemolysis were observed. This aligns with pharmacovigilance data indicating that IVIG is generally well-tolerated even in complex patients, supporting its feasibility for use in this high-risk population when managed with appropriate monitoring, consistent with expert recommendations for immunomodulatory therapies (16).

Exploratory subgroup analyses, though underpowered, showed consistent SOFA reduction favoring IVIG across all infection sources and pathogen types (differences ranging from +1.3 to +1.6 points), supporting the biological plausibility of the intervention.

The discrepancy between the chi-square (*p* = 0.043) and log-rank (*p* = 0.571) tests is explained by the timing of deaths: 71% of control deaths occurred within the first 10 days, while IVIG group deaths were evenly distributed. The log-rank test accounts for the entire survival timeline and is influenced by later curve convergence; with only 30 total events, the analysis lacks sufficient power for time-to-event detection.

### 4.1 Limitations and future directions

This study has limitations. Its single-center design may limit generalizability to other settings with different demographics, pathogens, or standards of care. The sample size (120 patients) was calculated to detect a 12% absolute mortality reduction with 80% power. Although the observed reduction (13.4%) exceeded this assumption, the chi-square *p*-value (0.043) only marginally crossed significance, the log-rank test was non-significant (*p* = 0.571), and the lower bound of the RR confidence interval (0.31) approaches a null effect. Thus, 120 patients may be insufficient for robust mortality conclusions in a heterogeneous sepsis population, and the observed benefit should be interpreted with caution. Subgroup analyses were not powered for statistical comparisons; therefore, these results are descriptive and hypothesis-generating only, and no statistical inferences should be drawn. The open-label design, despite blinded outcome assessment, carries a risk of performance bias. Follow-up was restricted to 28 days, precluding evaluation of important long-term outcomes including 90-day mortality, long-term functional status, and quality of life. These are significant limitations of this work. Regarding the discrepancy between chi-square (*p* = 0.043) and log-rank (*p* = 0.571) tests: 71% of control deaths occurred within the first 10 days, while IVIG group deaths were evenly distributed. The chi-square test detects this early separation, but the log-rank test accounts for the entire timeline and is influenced by later curve convergence. With only 30 total events, the study is underpowered for time-to-event analysis (>200 events needed for 80% power). The adjusted Cox model yielded HR 0.61 (95% CI 0.35–1.06; *p* = 0.081), confirming a trend without definitive significance. Thus, the survival benefit requires confirmation in larger studies.

Notable strengths include the prospective, randomized design; rigorous phenotyping of a specifically elderly sepsis cohort; use of validated organ dysfunction scores; and serial biomarker measurements that support a mechanistic link between immunomodulation and clinical improvement.

To advance these findings, a large, multicenter randomized study in elderly sepsis patients is crucial to confirm efficacy and define the optimal IVIG dosing and timing, as well as its potential synergistic role with other immunomodulatory adjuncts, a strategy showing promise in other contexts of severe, persistent infection (17–19). Subsequent research should evaluate cost-effectiveness, identify predictive biomarkers of response, and investigate the precise mechanisms by which IVIG modifies the aged immune response to infection.

## 5 Conclusion

In elderly patients with sepsis, adjunctive therapy with intravenous immunoglobulin was associated with reduced 28-day mortality, accelerated recovery of organ dysfunction, and more rapid resolution of systemic inflammation compared to conventional therapy alone. The treatment also reduced ICU resource utilization and demonstrated a favorable safety profile. While a statistically significant reduction in 28-day mortality was observed, the survival curve analysis showed a non-significant trend, highlighting the need for confirmatory studies. These findings suggest that IVIG may be a beneficial immunomodulatory adjunct in this high-risk, vulnerable population. Validation in larger, multicenter study is necessary to definitively establish efficacy, refine patient selection, and inform the integration of this approach into standard sepsis management protocols for the elderly.

## Data Availability

The original contributions presented in the study are included in the article/Supplementary material, further inquiries can be directed to the corresponding author.
