# Supplementary material for: Adjuvant intravenous immunoglobulin in elderly sepsis: a randomized controlled study of mortality, organ function, and inflammation
Source: Front Med (Lausanne). 2026 Jun 24;13:1857404. doi: 10.3389/fmed.2026.1857404 (PMC13342174; doi:10.3389/fmed.2026.1857404)
Supplement: Supplementary file 1 [file Data_Sheet_1.docx]

**Adjuvant Intravenous Immunoglobulin in Elderly Sepsis: A Randomized Controlled Study of Mortality, Organ Function, and Inflammation**

**Authors:** Xiaoyun Miao**^1^**, Jiaxin Shen**^1^**, Jinglin Zhao**^1^**, Rui Wang**^1^**, Hao Wang**^1^**, Qingchun Dai**^1^**^*^

**Address:**

**^1^** Department of Critical Care Medicine, Cangzhou Central Hospital, 061000, Cangzhou City, Hebei Province, China.

**Correspondence: *** Qingchun Dai**^1^**, **E-mail:** [qingchundai@ldy.edu.rs](mailto:qingchundai@ldy.edu.rs)

[ORCID-](https://orcid.org/0000-0002-7614-7397) 0009-0000-8476-6772

**Co-Authors:** Xiaoyun Miao**^1^**, E-mail: xiaoyun3870@163.com

Jiaxin Shen**^1^**, E-mail: 15831881111@163.com

Jinglin Zhao**^1^**, E-mail: sunday2252@163.com

Rui Wang**^1^**, E-mail: 13623170808@163.com

Hao Wang**^1^**, E-mail: xiaohao829@163.com

**Table S1.** Kinetics of inflammatory biomarkers during follow-up.

| **Parameter & Timepoint** | **IVIG Group (n=60)** | **Control Group (n=60)** | **P-value** |
| --- | --- | --- | --- |
| **C-reactive protein (mg/L)** |  |  |  |
| Baseline value, mean ± SD | 129.4 ± 22.7 | 131.1 ± 21.5 | 0.68 |
| Reduction from baseline to Day 3, mean ± SD | 32.5 ± 8.3 | 18.7 ± 7.5 | <0.001 |
| Reduction from baseline to Day 7, mean ± SD | 55.6 ± 10.4 | 38.3 ± 9.7 | <0.001 |
| **Procalcitonin (ng/mL)** |  |  |  |
| Baseline value, mean ± SD | 14.2 ± 3.8 | 13.9 ± 4.1 | 0.71 |
| Reduction from baseline to Day 3, mean ± SD | 2.8 ± 1.2 | 1.7 ± 1.0 | 0.020 |
| Reduction from baseline to Day 7, mean ± SD | 5.3 ± 1.6 | 3.1 ± 1.4 | <0.001 |

Data are presented as mean ± standard deviation. Reduction represents the absolute decrease from the baseline value. P-values are from independent samples t-tests comparing the magnitude of reduction between groups at each timepoint.

**
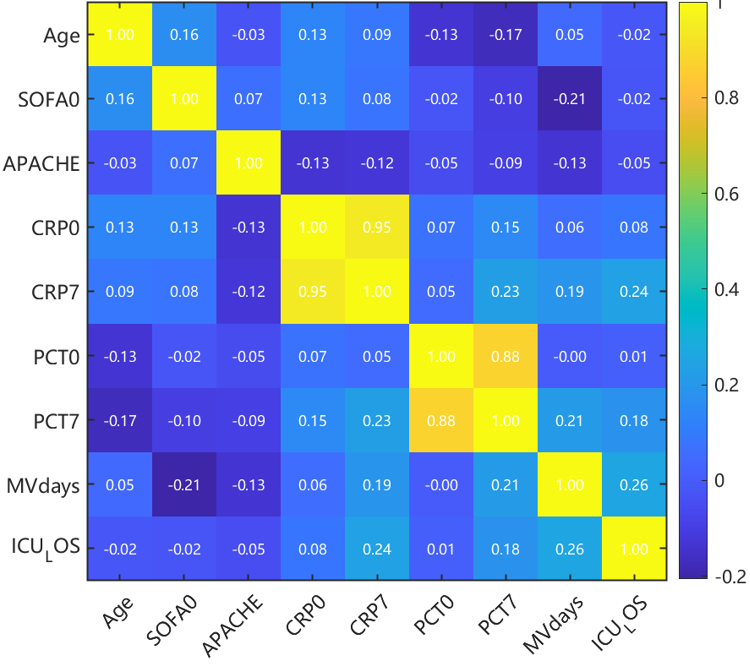
FIGURE S1.** Correlation matrix of continuous clinical variables. Heatmap showing pairwise Pearson correlation coefficients among age, baseline severity scores (SOFA, APACHE II), inflammatory markers (CRP, PCT), and resource use outcomes (mechanical ventilation days, ICU length of stay). Color intensity indicates correlation strength and direction.

**
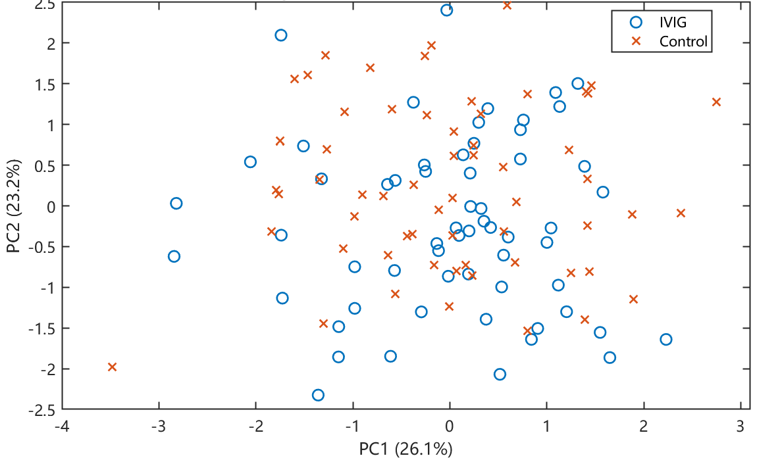
FIGURE S2.** Principal component analysis of baseline characteristics. Scatter plot showing the projection of patients from the IVIG and control groups onto the first two principal components derived from baseline variables (age, SOFA score, APACHE II score, C-reactive protein, and procalcitonin). Overlap between groups confirms balanced randomization across multiple dimensions.


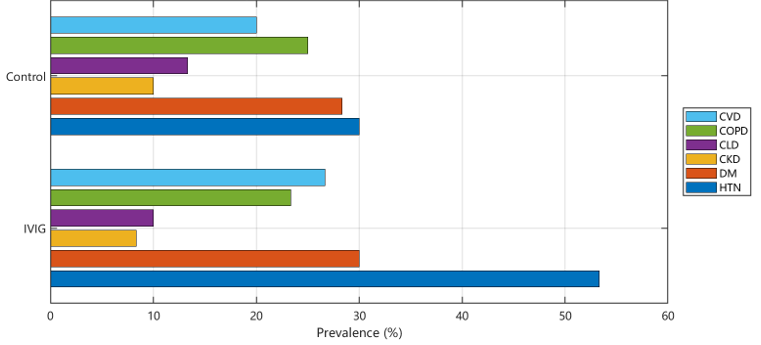
**
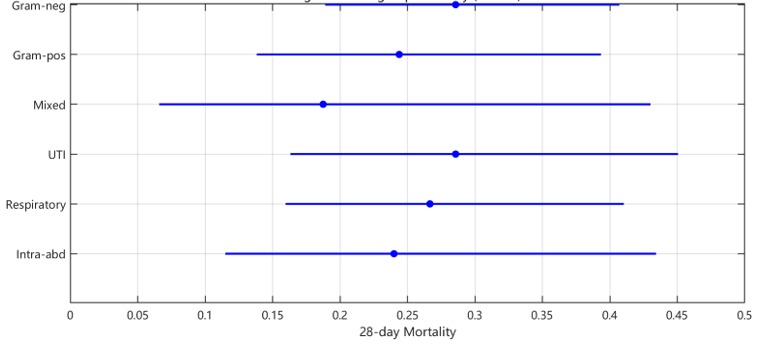
FIGURE S3.** Exploratory subgroup analysis of treatment effect on 28-day mortality. Forest plot illustrating mortality outcomes with intravenous immunoglobulin (IVIG) adjunctive therapy compared to conventional therapy alone across clinically relevant subgroups in elderly sepsis patients. Subgroups are based on pathogen type, infection source, and baseline severity. The plot shows point estimates (squares proportional to subgroup size) and 95% Wilson confidence intervals. Formal interaction tests were not performed due to limited sample size in subgroups.

**FIGURE S4.** Baseline comorbidity profiles. Horizontal bar chart illustrating the balanced distribution of major comorbidities, including hypertension, diabetes, chronic kidney disease, chronic obstructive pulmonary disease, and cardiovascular disease, between the treatment groups prior to intervention.


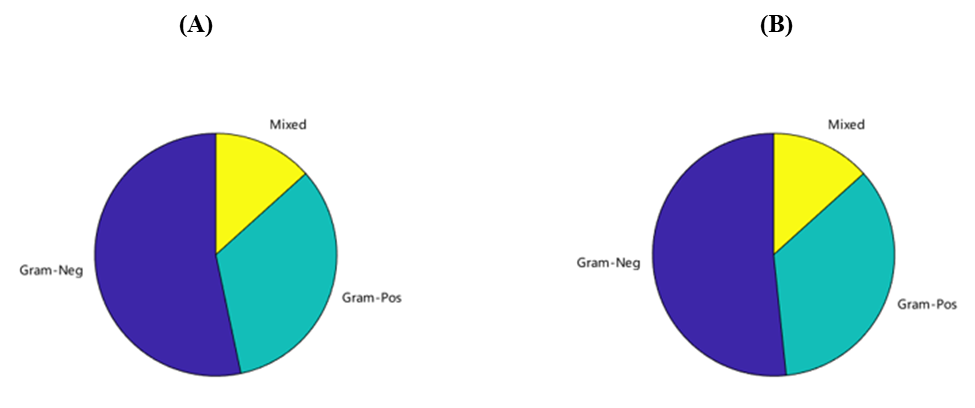


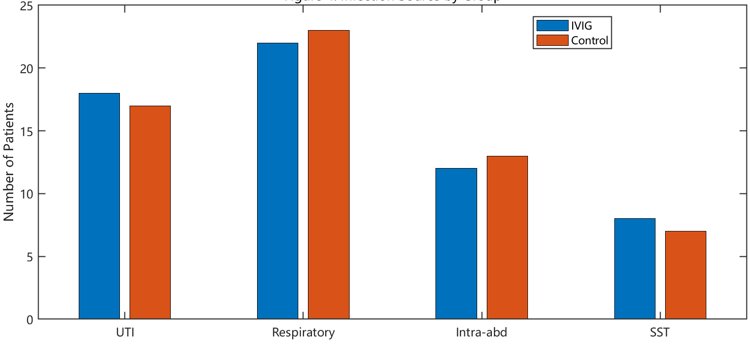
**FIGURE S5.** Distribution of causative pathogens by treatment group. Pie charts showing the proportion of Gram-negative, Gram-positive, and polymicrobial infections in (A) the intravenous immunoglobulin (IVIG) group and (B) the control group. Percentages are based on culture-confirmed infections (IVIG: n=60; Control: n=60). The distribution did not differ significantly between groups (p=0.85).

**FIGURE S6.** Distribution of primary infection sources. Clustered bar chart showing the number of patients with urinary tract, respiratory, intra-abdominal, and skin/soft tissue infections in the intravenous immunoglobulin (IVIG) and control groups. Distributions were comparable between groups (p>0.78 for all pairwise comparisons by chi-square test).

**
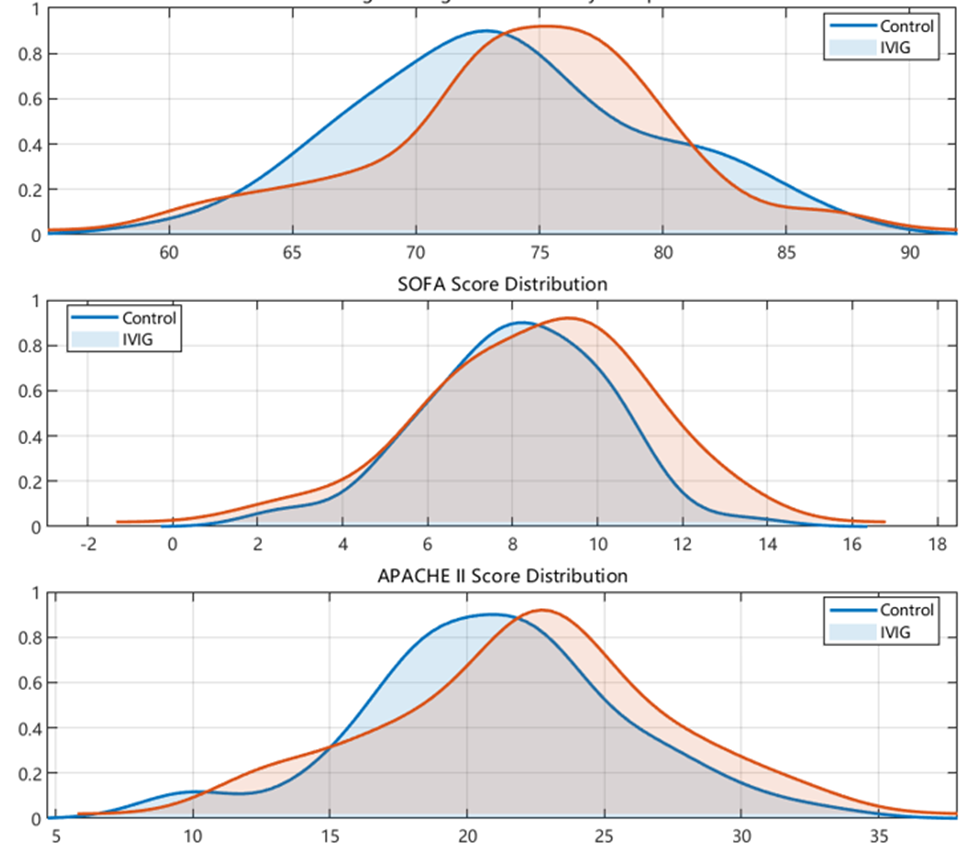
FIGURE S7.** Distribution of baseline characteristics. Kernel density plots showing the distribution of (**A**) age, (**B**) baseline SOFA score, and (**C**) baseline APACHE II score for patients in the intravenous immunoglobulin (IVIG) and control groups.
